# Comparative Effectiveness and Safety of Direct Oral Anticoagulants in Low Body Weight Patients with Atrial Fibrillation: A Systematic Review and Meta-analysis

**DOI:** 10.1007/s10557-023-07537-x

**Published:** 2024-01-02

**Authors:** Mohamed Nabil Elshafei, Muhammad Salem, Ahmed El-Bardissy, Mohamed S. Abdelmoneim, Ahmed Khalil, Sherine Elhadad, Mutasem Al Mistarihi, Mohammed Danjuma

**Affiliations:** 1 Clinical Pharmacy Department, Hamad Medical Corporation, Doha, Qatar; 2 Pharmacy department, The View Hospital, Doha, Qatar; 3 Internal Medicine Department, Hamad Medical Corporation, Doha, Qatar; 4 Weill Cornell Medicine-Qatar, Doha, Qatar

**Keywords:** Atrial fibrillation, Warfarin, DOAC, Low body weight, Meta-analysis

## Abstract

**Introduction:**

Direct oral anticoagulant (DOAC) agents are established as the anticoagulation strategy of choice for a variety of clinical risks. Despite this, uncertainty still exists with regard to their efficacy and safety for the prevention of stroke and systemic embolism in some patient populations; most notably those with low body weight (LBW) (<60 kg or body mass index [BMI] <18 kg/m^2^). Currently, there is a paucity of trial and non-trial data to support a prescriptive recommendation for their use in these patient cohorts. We have carried out a pooled systematic review of the most up to date published data of patients stabilized on various DOAC analogs with the view to ascertaining the exact matrices of their efficacy and safety in these cohorts of patients.

**Methods:**

We initially carried out a comprehensive search of databases from inception to June 2023 for eligible studies exploring the efficacy and safety of various analogs of direct oral anticoagulants in patients with atrial fibrillation who had low body weight. Databases accessed include PubMed, EMBASE, the Science Citation Index, the Cochrane Database of Systematic Reviews, and the Database of Abstracts of Reviews of Effectiveness. We carried out a weighted comparison of derived pooled odd ratios (with their corresponding confidence intervals) of mortality outcomes between various DOACs using the random effects model.

**Results:**

Thirteen studies (n = 165,205 patients) were included in our meta-analysis. DOAC analogs were associated with increased stroke-related events, composite outcome, and mortality in low body weight patients compared to non-low body weight patients (odds ratio [OR] 1.50, 95% confidence interval [CI] 1.17–1.92), (OR 1.55, 95% CI 1.29–1.86), (OR 2.92, 95% CI 1.87–4.58), respectively. There was no significant difference in the safety outcome (major bleeding events) between the DOAC analogs (OR 1.19, 95% CI 0.93–1.52).

**Discussion:**

In this meta-analytical review comprising both real-world and randomized controlled studies, the use of DOAC analogs in low body weight patients (body weight of <60 kg or BMI<18 kg/m^2^) with atrial fibrillation was associated with increased risks of stroke-related events, composite outcomes, and mortality compared to non-low body weight cohorts patients. At the same time, there was no significant difference in terms of major bleeding events. This finding has provided the first resolution of pervading uncertainty surrounding the use of DOAC analogs in these patient cohorts and suggests the need for follow-up confirmatory systematic studies in this group of patients.

## Introduction

Atrial fibrillation (AF) is a cardiac conduction abnormality characterized by irregular heart rhythm, which disrupts atrial coordinated contraction resulting in increased risk of thrombus formation and ischemic stroke [1]. It is the most common cardiac arrhythmia in clinical practice [2]. AF patients are at enhanced risk for death, heart failure, hospitalization, and thromboembolic events [2–4]. Anticoagulation is mandatory to mitigate the multiplicative risks of ischemic stroke in AF patients [1]. Until a few years ago, initially bridging with low-molecular-weight heparin (LMWH) followed by oral anticoagulation (OAC) with vitamin K antagonists (VKA) was considered the standard treatment approach [5]. However, with the emergence of pharmacokinetic (PK) and pharmacodynamic problems with warfarin such as logistics of international normalized ratio (INR) monitoring, predisposition to various bidirectional drug–drug interactions, as well as inter and intra-individual variability in its PK profiles necessitated the search for alternative anticoagulation strategies. [6–9]. The direct oral anticoagulants (DOACs), including factor IIa (thrombin) and factor Xa inhibitors, have been developed and introduced to the market. They are approved by the Food and Drug Administration (FDA) for stroke prevention in AF patients [10–13]. A continuous stream of randomized controlled clinical trials has shown that these agents were non-inferior compared to VKA in terms of both efficacy and safety in reducing the risk of stroke and systemic embolization in patients with AF [14–18]. Consequently, they have been integrated into both national and society-based therapeutic guidelines [5]. Since marketing authorization, DOACs have significantly changed the landscape of AC management, with current guidelines recommending their use over warfarin in non-valvular AF [5, 19].

Despite the widely reported favorable fixed dosing therapeutic characteristics of DOACs (including fewer food and drug interactions and lack of need for therapeutic drug monitoring among others), concerns have continued unabated regarding their use in therapeutically challenged patient populations such as those with LBW [20]. The limited inclusion of LBW patients in the determinant major trials of DOACs has added to these concerns, especially regarding their efficacy, adequacy of fixed dosing, and overall safety profile in these patient populations. The paucity of evidence from studies investigating the efficacy and safety of DOACs in low body weight patients has compounded the resolution of these concerns. From the few studies that have evaluated this, outcomes with regard to both their efficacy and safety in low body weight patients have been discordant. For example, in the adjusted analyses of a recent Apixaban study, the use of Apixaban in LBW patients was not associated with higher rates of bleeding or thrombotic events, compared to cohorts with normal body weight [21]. Conversely, a sub-analysis of a Japanese population study with non-valvular atrial fibrillation showed that underweight patients had higher incidence of thromboembolic events and all-cause mortality in patients compared to those with normal body weight [22].

Pharmacokinetic studies of DOACs revealed that drug levels are influenced by factors such as age, weight, kidney function, and concurrent medications [23, 24]. The impact of DOACs is attributed to their plasma concentration, which can be influenced by the volume of distribution within the body. Extreme variations in body weight have the potential to affect the efficacy and safety of DOACs.

This, therefore, suggests that there is an unresolved uncertainty regarding the utility of DOAC analogs as stroke prevention AC strategy in low body weight patients (BMI <18 kg/m^2^ or weight <60 kg). The irreducible minimum as far as stroke prevention and reduction of systemic embolization in these cohorts of patients is to establish that DOACs were at least non-inferior to VKA in terms of efficacy and safety.

This meta-analysis, therefore, aimed to evaluate the effectiveness (rates of stroke events, composite outcome, mortality), and safety (major bleeding) of DOAC analogs in patients with extremely LBW with AF. This is with the view to providing additional clarity and resolve lingering uncertainty regarding the use of these agents in these cohorts of patients.

## Methods

This review followed PRISMA guidelines [25].

### Study Eligibility Criteria

We include both real-world observational data and randomized controlled trials that compared DOAC analogs in LBW patients (BMI <18 or weight <60 Kg). We required that at a minimum, studies must assess stroke recurrence or major bleeding events to be included in the review. We excluded studies reporting on pediatrics (<18 years old), as well as studies failing to meet the inclusion criteria.

### Search Strategy

We performed an exhaustive literature search of PubMed, Medline, and EMBASE since their inception till 01/06/2023 (June 2023). No language, date, or article type limitations were adopted in our search strategy. Example of a database search strategy is: (((((((((((((((((NOACS) OR (DOACS)) OR (new oral anticoagulants)) OR (rivaroxaban)) OR (dabigatran)) OR (apixaban)) OR (edoxaban)) OR (anticoagulant agents[MeSH Terms])) OR (NOACS[MeSH Terms])) OR (DOACS[MeSH Terms])) OR (NOACS[Title/Abstract])) OR (DOACS[Title/Abstract])) OR (Apixaban[Title/Abstract])) OR (rivaroxaban[Title/Abstract])) OR (dabigatran[Title/Abstract])) OR (edoxaban[Title/Abstract]) AND (2022/5/31:2023/5/10[pdat])) AND ((((((((low body weight[Title/Abstract]) OR (low body-weight[Title/Abstract])) OR (LBW[Title/Abstract])) OR (low body weight)) OR (underweight)) OR (low body-weight)) OR (low body weight[MeSH Terms])) OR (under weight[MeSH Terms]) AND (2022/5/31:2023/5/10[pdat]))) AND (((((((atrial fibrillation) OR (AF)) OR (atrial fibrillation[MeSH Terms])) OR (atrial fibrillations[MeSH Terms])) OR (atrial fibrillation[Title/Abstract])) OR (AF[Title/Abstract])) OR (AFib[Title/Abstract]) AND (2022/5/31:2023/5/10[pdat])). Additionally, we attempted a manual reference search of retrieved studies.

### Screening and Data Extraction

Following initial title and abstract screening, we retrieved eligible articles for a full-text review and assessment for inclusion in the review. Two reviewers (MNE & MAR) conducted the search and screening. In the case of disagreement this was resolved by consensus, and rarely a third reviewer (MSA) adjudicates when consensus fails. We initially trialed a data extraction form on a few articles to test its validity before applying it to the other included studies. Examples of the data extracted are general article information such as the author, publication year, study design, intervention, control, outcome, weight, etc.

### Review Outcomes

The primary outcome of the review is the rate of stroke recurrence, composite outcome, and all-cause mortality. Major bleeding events were designated as the review’s secondary outcome (as defined by the primary study authors). We would look at these outcomes at 6 months of follow-up whenever specified in the study, otherwise we considered the longer duration of observation if no specification was provided by the study investigators.

### Study Quality and Risk of Bias Assessment

Using the Cochrane Risk of Bias Tool for Randomized Controlled Trials, reviewers evaluated the risk of bias (ROB) in the included studies [26]. The six bias domains addressed by the risk of bias tool are selection bias, performance bias, detection bias, attrition bias, reporting bias, and other biases. Cohort study quality was evaluated using the Newcastle Ottawa Scale (NOS) [27]. Eight fundamental factors were measured using the NOS, broken into three major categories: comparability, exposure, and research quality selection. In the case of post hoc analysis, we also took a distinct strategy to evaluate the risks of bias in each of the original trials while using data from the research [28]. The Review Manager (RevMan) software version 5.4 and the Risk-of-Bias Visualization (robvis) tools were used to create the visualization of the ROBs numbers.

### Statistical Analysis

The odds ratios (OR) were computed as a measure of effect size. The Forest plot was generated to summarize the results. Additionally, we conducted a sensitivity analysis to screen for consistency and small-study effects. The *I*^*2*^ statistic was used to report heterogeneity. An *I*^*2*^ >50% is suggestive of marked heterogeneity in our review. The random-effects model was used as our meta-analytical technique. STATA software was used for statistical analysis (Stata MP 15 (StataCorp, College Station, TX).

## Results

**Fig. 1:**
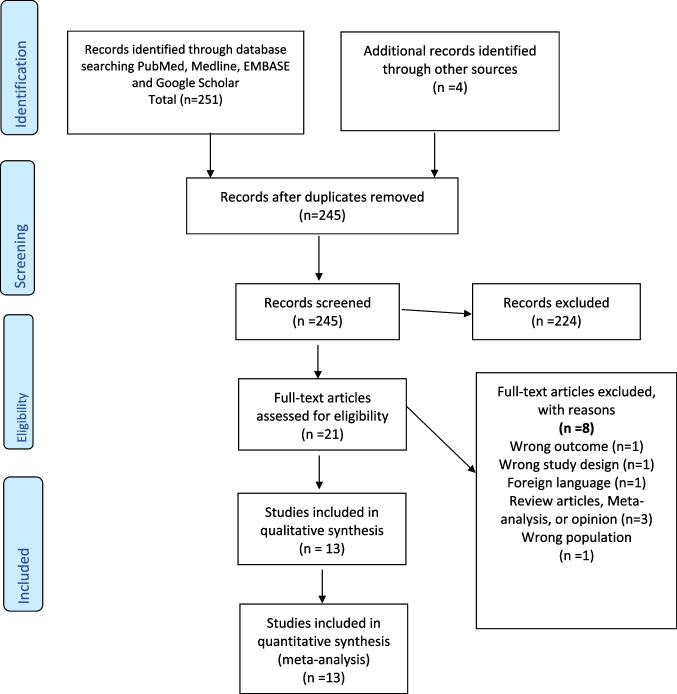
PRISMA flow diagram

Our search strategy retrieved 251 titles. After screening, 13 studies were included in our final analysis (Fig. 1 shows the PRISMA flow diagram) [23, 24, 28–37, 39]. The total number of patients evaluated in these studies is 165,205 patients. The included studies were observational and randomized controlled studies meeting our eligibility criteria. (Table 1 summary of studies included in the meta-analysis).

**Table 1:** Characteristics of AF studies (n = 13)

Study author	Country	Design	Anticoagulant, *n* (%)	LBW and Non LBW (*n*)	Mean age, years ± SD, or median, years (IQR)	Male, %	Patients included	Weight-based reported efficacy outcomes	Safety outcomes	Follow up time
Boehringer Ingelheim (RE-LY Dabi-150) 2009 [10, 29]	Global	RCT	Dabigatran-150, 6076 (50) Warfarin, 6022 (50)	LBW: 1331 Non LBW: 10762 LBW Dabigatran 150: 647 LBW Warfarin: 684	Dabigatran 150: 71.5 ± 8.8 Warfarin: 71.6 ± 8.6	Dabigatran 150: 63.2 Warfarin: 63.3	NVAF	IS, systemic embolism	Major bleeding	24 M
Bayer AG (ROCKET AF) 2011 [11, 30]	Global	RCT	Rivaroxaban, 7131 (50) Warfarin, 7133 (50)	LBW: 1555 Non LBW: 12709 LBW Rivaroxaban: 777 LBW Warfarin: 778	Rivaroxaban: 73 (65–78) Warfarin: 73 (65–78)	Rivaroxaban: 60.3 Warfarin: 60.3	NVAF	IS, systemic embolism	Major bleeding	24 M
Daiicii Sankyo (ENGAGE AF-TIMI 48) (high dose Edox) 2013 [12, 31]	Global	RCT	High-dose Edoxaban, (7035) Warfarin, (7036)	LBW: 1385 Non LBW: 12686 LBW HD-Edoxaban: 684 LBW Warfarin: 701	HD-Edoxaban: 72 (64–78) Warfarin: 72 (64–78)	HD-Edoxaban: 62.1 Warfarin: 62.5	NVAF	IS, systemic embolism	Major bleeding	33.6 M
Park et al 2017 [32]	South Korea	Retrospective cohort	Apixaban, 244 (18). Dabigatran, 620 (45.8). Rivaroxaban, 489 (36.1).	LBW: 62 Non LBW: 1291	LBW: 77.4 ± 7.1 Non LBW: 72.3	LBW: 51.6 Non LBW: 53.1	AF	IS, systemic embolism, all-cause mortality	Major bleeding	7 M
Hohnloser et al (ARISTOTLE) 2019 [13, 33]	Global	RCT	Apixaban, 9088 (50.1) Warfarin, 9051 (49.9)	LBW: 1985 Non LBW: 16154 LBW Apixaban: 1018 LBW Warfarin: 967	LBW: 74 (66–79) Non LBW: 66 Apixaban: 70 (63–76) Warfarin: 70 (63–76)	LBW: 28 Non LBW: 69.3 Apixaban: 64.5 Warfarin: 65	NVAF	IS, systemic embolism, MI, all-cause mortality	Major bleeding	21.8 M
Murakawa et al (XAPASS) 2020 [22]	Japan	Prospective cohort	Rivaroxaban, 7618 (100)	LBW: 542 Non LBW: 7076	LBW: 78.1 ± 9.0 Non LBW: 71.5	LBW: 47 Non LBW: 63.8	NVAF	Stroke, non-CNS systemic embolism, MI, all-cause mortality	Major bleeding	12 M
Boriani et al (ETNA-AF-Europe) 2021 [34]	Europe	Prospective cohort	Edoxaban, 12667 (100)	LBW: 1310 Non LBW: 11,357	LBW: 78.4 ± 8.64 Non LBW: 71.4	LBW: 12.6 Non LBW: 61.8	NVAF	Stroke, systemic embolism, MI, all-cause mortality	Major bleeding	12 M
Kadosaka et al (J-ELD AF) 2021 [35]	Japan	Prospective cohort	Apixaban, 3025 (100)	LBW: 2006 Non LBW: 1019	LBW: 82.8 Non LBW: 79.7 ± 3.9	LBW: 37.7 Non LBW: 79.6	NVAF	Stroke, systemic embolism, all-cause mortality	Major bleeding	12 M
Lee et al 2021 [36]	South Korea	Retrospective cohort	Apixaban, 8489 (25.2) Dabigatran, 7020 (20.8) Edoxaban, 5907 (17.5) Rivaroxaban, 12261 (36.4) Warfarin, 9496 (22.0)	LBW: 1154 Non LBW: 42019	LBW: 76.6 ± 10.1 Non LBW: 70.6	LBW: 54.3 Non LBW: 60	NVAF	IS, all-cause mortality	Major bleeding	7.2 M
Bodega et al (INSIghT) 2022 [37]	Italy	Prospective cohort	Apixaban, 245 (30.2) Dabigatran, 220 (27.1) Edoxaban, 155 (19.1) Rivaroxaban, 192 (23.6)	LBW: 108 Non LBW: 704	LBW: 78 ± 9.3 Non LBW: 65.3	LBW: 12 Non LBW: 28.9	NVAF	IS/TIA, systemic embolism, MI, all-cause mortality	Major bleeding	24 M
Ishii et al (AFIRE) 2022 [38]	Japan	Post hoc analysis of RCT	Rivaroxaban, 2054 (100)	LBW: 72 Non LBW: 1982	LBW: 78.5 (72.3-83) Non LBW: 73	LBW: 60 Non LBW: 80	NVAF	Stroke, MI, systemic embolism, unstable angina requiring revascularization, all-cause mortality	Major bleeding	45 M
Nakao et al 2022 [39]	UK	Retrospective cohort	Apixaban, 2617 (9.0) Dabigatran, 579 (2.0) Edoxaban, 151 (0.5) Rivaroxaban, 2970 (9.8) Warfarin, 22,818 (78.3)	LBW: 585 Non LBW: 28550	LBW: 83.6 Non LBW: 77.3	LBW: 31.2 Non LBW: 54.2	AF	IS, all-cause mortality	Major bleeding	44.4 M
DeCamillo et al 2023 [21]	USA	Retrospective cohort	Apixaban, 545 (100)	LBW: 78 Non LBW: 467	LBW: 79.5 ± 9.5 Non LBW: 74.6 ± 10.0	LBW: 6.4 Non LBW: 46	NVAF	IS/TIA	Major bleeding	12 M

*LBW*, low body weight; *NAVF*, non valvular atrial fibrillation; *IS*, ischemic stroke; *HD*, high dose

### Recurrent Stroke

**Fig. 2:**
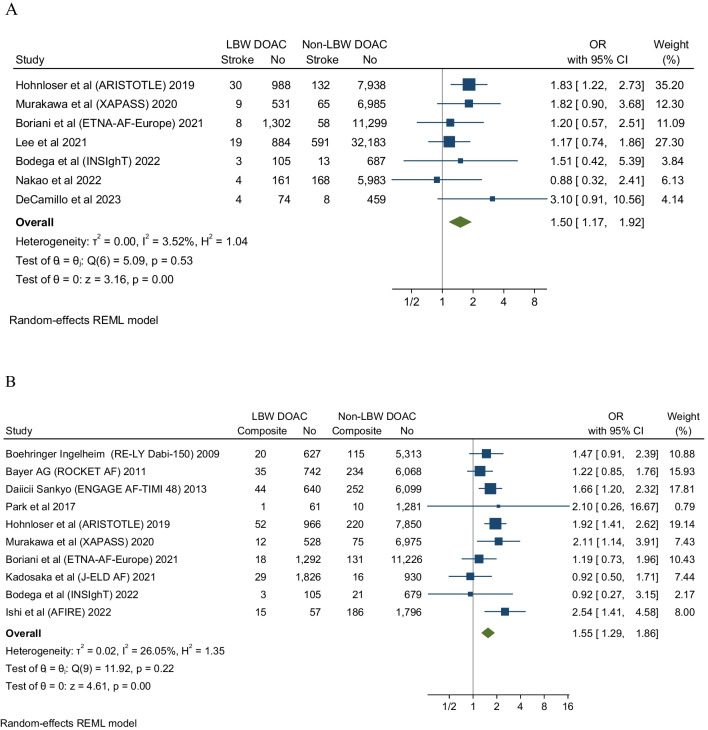

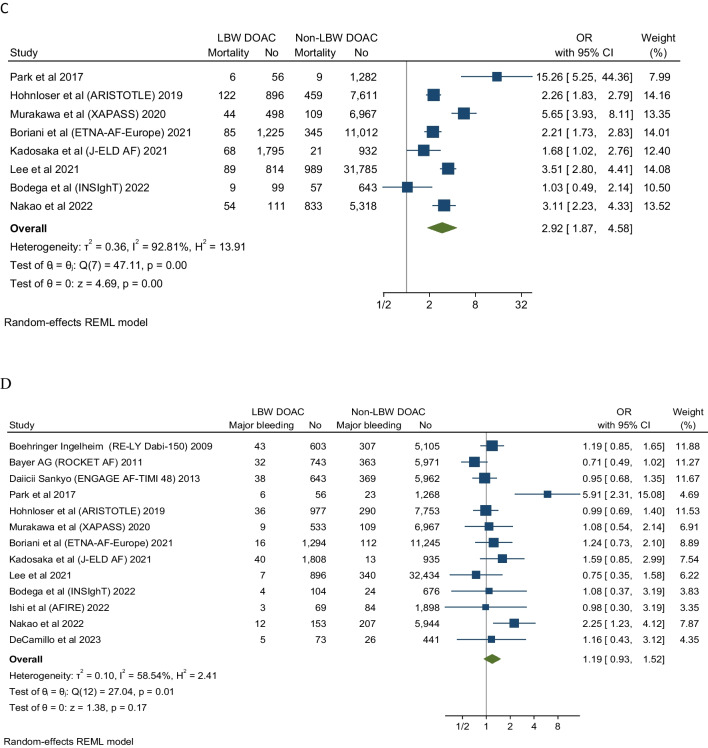
**A** Depicting a forest plot of stroke recurrence rates in DOAC analogs in LBW patients compared to non-LBW patients. **B** Depicting a forest plot of composite outcomes in DOAC analogs in LBW patients compared to non-LBW patients. **C** Depicting a forest plot of Mortality in DOAC analogs in LBW patients compared to non-LBW patients. **D** Depicting a forest plot of major bleeding events in DOAC analogs in LBW patients compared to non-LBW patients

**Fig. 3:**
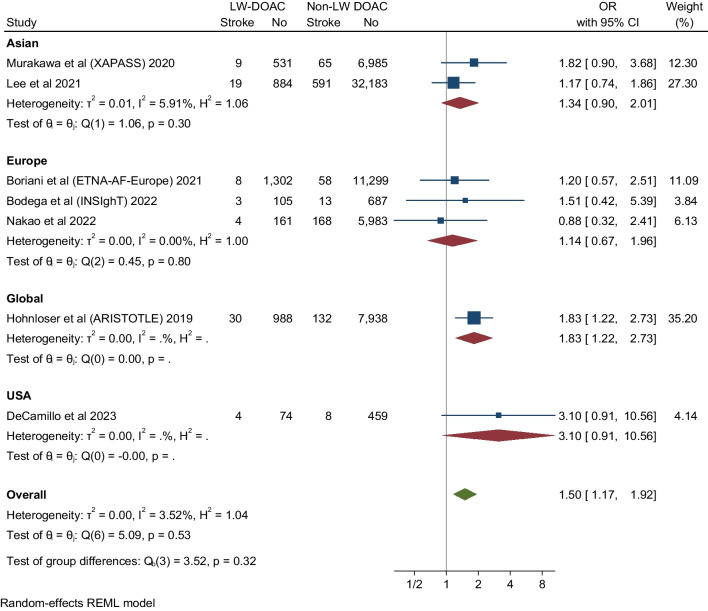
Subgroup analysis assessing stroke events in DOAC in LBW vs. Non-LBW patients according to ethnicity

**Fig. 4:**
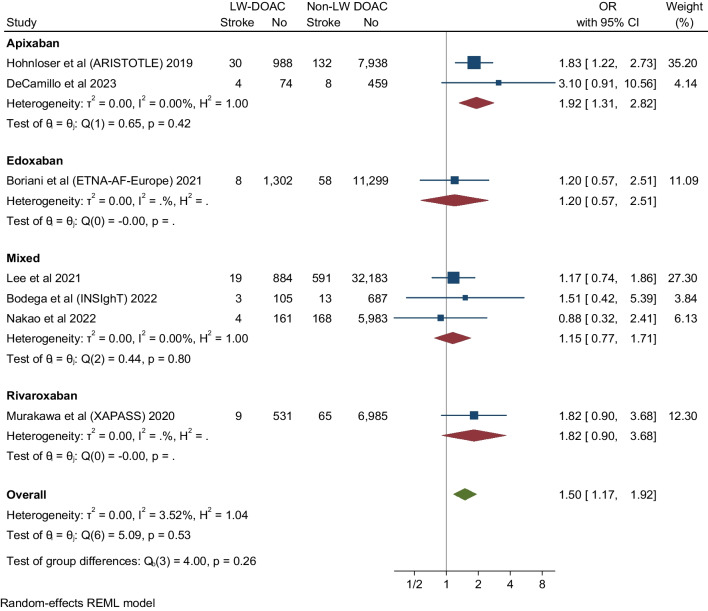
Subgroup analysis assessing stroke events in DOAC in LBW vs Non-LBW patients according to type of DOAC

**Fig. 5:**
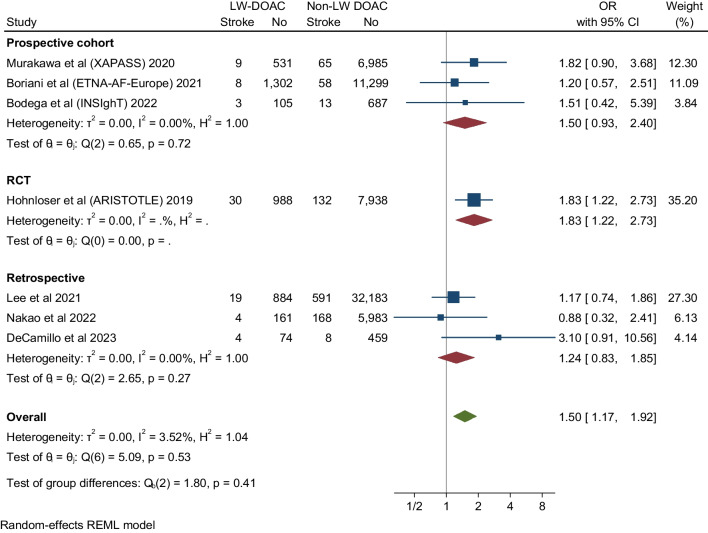
Subgroup analysis assessing stroke events in DOAC in LBW vs Non-LBW patients according to study type

**Fig. 6:**
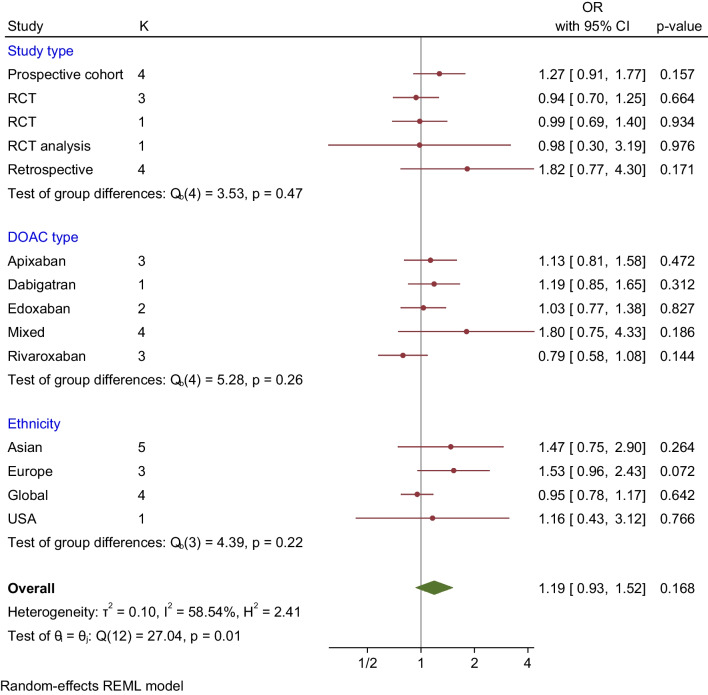
**A** Funnel plot to assess the publication bias for studies assessing stroke recurrence in DOAC in LBW vs Non-LBW patients displaying no marked asymmetry. **B** Funnel plot to assess the publication bias for studies assessing major bleeding events in DOAC in LBW vs Non-LBW patients showing no marked asymmetry

Seven studies evaluated stroke recurrent events in low body weight patients. These studies showed that DOAC analogs were associated with about a 50% increase of stroke events in low body weight patients compared to non-low body weight patients (OR 1.5, 95% CI 1.17-1.92), Q 5.09, I2 3.52%. The low *I*^*2*^ inferring low homogeneity suggests a degree of certainty around the point estimates this outcome (Fig. 2). The funnel plot revealed no marked asymmetry (Fig. 6). In subgroup analyses of the primary efficacy end point (Figs 3, 4 and 5) there was no significant differential effect of various ethnic compositions or individual DOAC analogs on the stroke endpoint (Fig. 3). Among all DOACS, only Apixaban demonstrated a significant increase in stroke events in LBW compared to non-LBW patients (Fig. 4).

### Composite Outcome

Ten studies reported composite outcome in low body weight patients. Prescribing DOAC in LBW patients with atrial fibrillation showed a trend toward an overall increase in composite outcome by 55% compared to non-low body weight patients or be it with uncertainty around the point estimate (OR 1.55, 95% CI 1.29–1.86, Q = 11.92, I 2 = 26.05%) (Fig. 2). The low I^2^ suggested the homogeneity of the results.

### Mortality

Eight studies evaluated mortality outcome in low body weight patients. These studies showed significant differences in mortality associated with DOAC use in LBW patients compared to non-LBW patients. In the pooled analysis of the eight studies, low body weight patients with AF who received DOAC had significant mortality difference versus non-low body weight patients (OR 2.92, 95% CI 1.87–4.58) (Fig. 2), Q = 47.11, I2 = 92.81%; however, there is significant heterogeneity among the studies.

### Major Bleeding

**Fig. 7:**
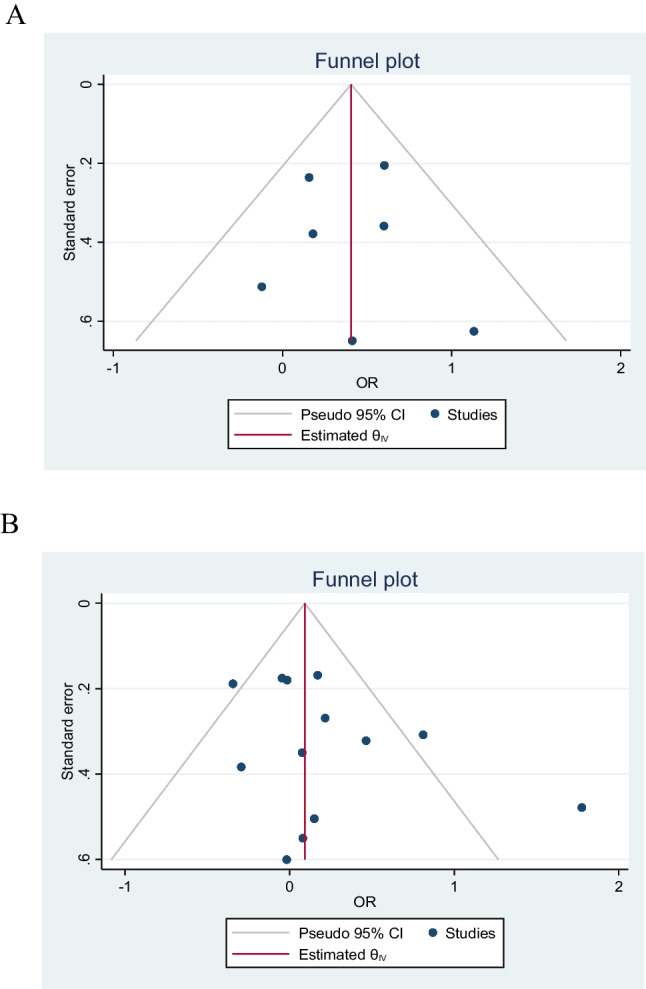
Subgroup analysis assessing major bleeding events in DOAC in LBW vs Non-LBW patients according to study type, DOAC type and ethnicity

Thirteen studies evaluated and reported the risk of major bleeding events. These studies showed that DOAC use among LBW patients had no significant difference in the major bleeding events compared to non-low body weight patients who used DOAC (OR 1.19, 95% CI 0.93-1.52, Q = 27.04, I2 = 58.54%) (Fig. 2). The funnel plot showed no marked asymmetry; however, there is significant heterogeneity among the studies (Fig. 6). We performed subgroup analysis according to DOAC type, ethnicity, and study type (Fig. 7).

### Risk of Bias Assessment

**Fig. 8:**
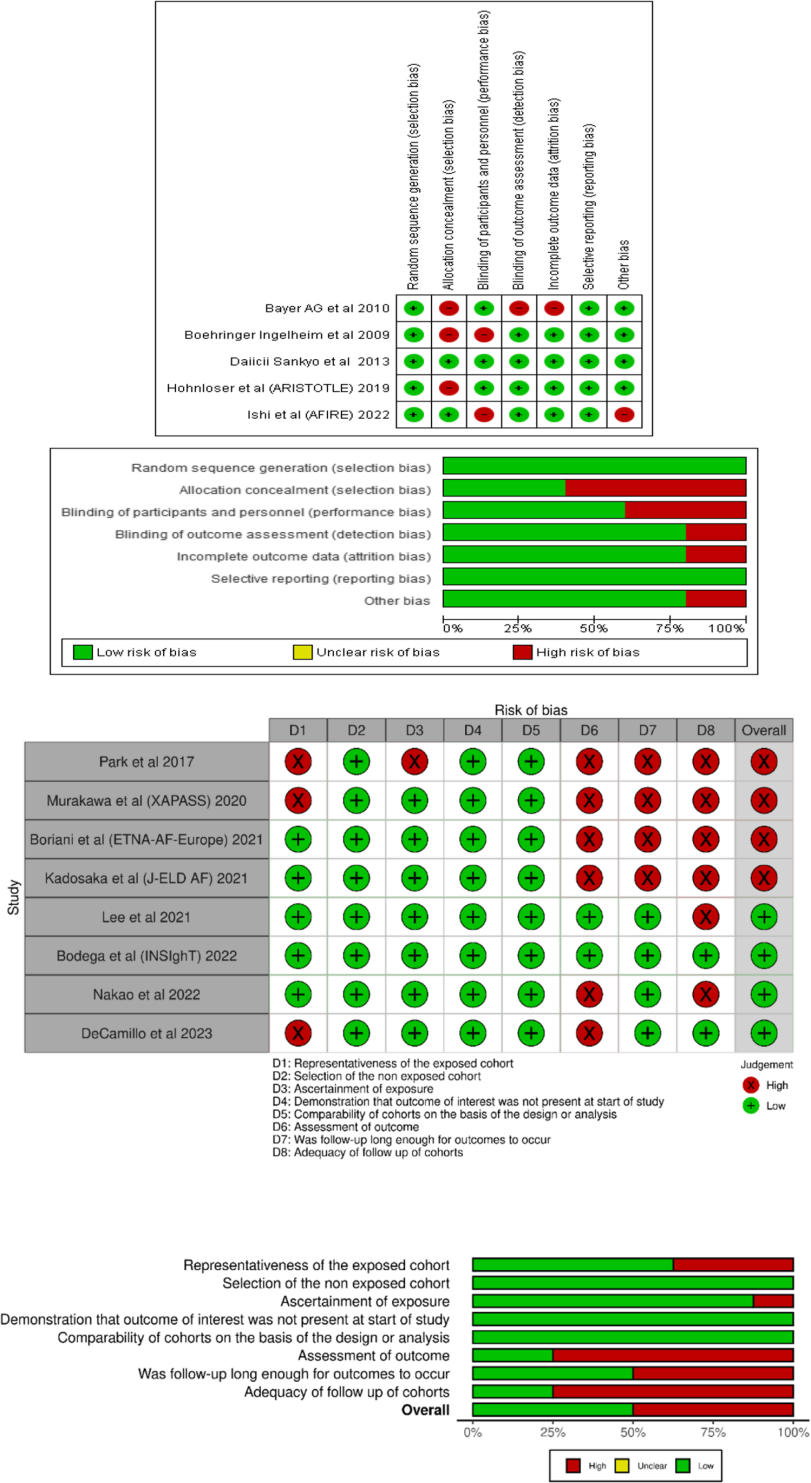
Risk of bias assessment

A total of three clinical trials and two post hoc analyses showed a low risk of bias (Fig. 8). The main domain of high risk among the five studies was allocation concealment (selection bias), followed by blinding of participants and personnel (performance bias).

The overall quality assessment of cohort studies showed that half of the studies had a low risk of bias, and the other half had a high risk of bias (Fig. 8). The “assessment of outcome” and “adequacy of follow-up of cohort” items were the leading causes of the high risk of bias (Fig. 8).

## Discussion

In this comprehensive pooled examination of pivotal studies, we investigated the impact of LBW in hard clinical endpoints in patients treated with various analogs of direct oral anticoagulants (DOACs) for stroke prevention in AF patients. Our synthesis showed that patients with LBW had an approximately 50% increased risk of stroke-related outcomes compared to non-low body weight patients (odds ratio [OR] 1.5, 95% confidence interval [CI] 1.17-1.92, p-value < 0.05). This represents a timely addition to our current understanding of the effect of this key variable on some of the most significant contributors to morbidity and mortality in these patients. Both pharmacokinetic and clinical studies have consistently identified LBW as an important criterion for dose adjustment of DOACs in patients stabilized on them for indications (ranging from AF through to venous thromboembolism). Despite initial uncertainties, recent prescriptive guideline recommendations have been more explicit. For example, for Dabigatran and Rivaroxaban, dose adjustments based on LBW are not recommended [40, 41]. Conversely, apart from LBW (≤60 kg), patients stabilized or about to go on Apixaban require additional criteria for dose adjustment, including serum creatinine ≥1.5 mg/dL or age ≥80 years [40, 41].

Additionally, our pooled synthesis of the eight studies that evaluated mortality outcomes in LBW patients showed a significant difference in mortality associated with DOAC use in LBW patients compared to non-low body weight patients. In the pooled analysis of the eight studies, LBW patients with AF who received DOAC had significant mortality difference versus non-low body weight patients (OR 2.92, 95% CI 1.87–4.58) (Fig. 2), Q = 47.11, I2 = 92.81%; however, there was significant heterogeneity among the constituent studies. Earlier studies have already suggested increased risks of excess mortality (due to inappropriate underdosing) among patients on DOACs. Following initial marketing authorization of DOACs, among a whole range of potential “reliefs” they were forecasted to usher in was the lack of need for therapeutic drug monitoring; and on this count alone, they were judged to have a wider therapeutic index compared to standard vitamin K antagonists [42]. However, subsequent emergence of weight considerations has raised potential concerns around whether these drugs truly do have wide therapeutic indices. For patient cohorts that are overweight (weight >120 kg or BMI >40), a recent meta-analysis [Elshafei et al], and subsequent prescriptive recommended by the Internal Society for Thrombosis and Hemostasis (ISTH) have clarified the lack of concern regarding efficacy and untoward safety issues around these weight ranges [43]. For cohorts with LBW and for all indications, unfortunately, uncertainty still abounds, especially around the binary outcomes of overdosing and underdosing. Previous studies have already established that inappropriate underdosing may be associated with increased risks of strokes [42, 44, 45] and overdosing with a range of adverse effects profiles, including major and minor bleeding. The combined findings from our synthesis (increased risk of strokes, mortality, and composite outcomes) do suggest that for LBW, DOACs do have a narrow therapeutic index. However, unlike VKA’s, there are yet any well-validated pharmacokinetic measures or indexes to guide clinical care based on LBW (other than prescriptive recommendations by various national and international treatment guidelines.

Registry data from South Korea have suggested favorable efficacy and safety outcomes for both AF patients with low ((<60 kg) or very low body weight (<50 kg) [44]. The demography of the patient population within this registry is older (>70 years) with multi-morbidity; this is unlikely to represent the typical phenotype of patients in both Europe and North America. Body weight significantly influences the volume of distribution of drugs, a key. It must be emphasized that this study is limited by the relatively low number of patients on Edoxaban enrolled (n = 775 [8%]) [46]. It is pertinent to note that Edoxaban is one of the two DOACs with subsisting uncertainty regarding the exact effect of LBW on hard clinical endpoints (including mortality and bleeding risks) in patients exposed to them. Additionally, this registry data comes from a region where LBW is very common and an integral part of the population with all the necessary “Pharmacokinetic adjustments” in place. It is unlikely that the outcomes they have reported will hold true for other populations whose body weight dispositions are at the higher percentile of the general population.

Ethnicity is a key determinant of the pharmacokinetic variability seen in patients on DOACs and other drugs in general [47]. Indeed, this has extensively been studied in AF patients on DOACs in the US. In an examination of 69,553 patients hospitalized with AF from 159 sites between 2014 and 2020 and domiciled in the GWTG-AFIB registry, Essien et al. found that the bleeding risks (adjusted hazard ratio [aHR], 2.08; 95% CI, 1.53-2.83), stroke risks (aHR, 2.07; 95% CI, 1.34-3.20), and mortality risks (aHR, 1.22; 95% CI, 1.02-1.47) were all higher in Black patient cohorts than their White counterparts, and Hispanic patients had higher stroke risk (aHR, 2.02; 95% CI, 1.38-2.95) than White patients [48]. A sub-group analysis of our review population based on ethnic extraction showed no significant differential effect of various ethnic compositions (Asian, European, American) on the point estimate of the safety and efficacy outcomes tested. This will probably need further exploration as the variability between previous studies reporting no effect of LBW on DOAC-related outcomes and those reporting adverse outcomes has been attributable partially to the population where those studies were conducted. The Korean registry database report is a case in point [46].

Analyses of the effect of individual DOAC analogs on the risks of strokes showed instability of their various point estimates; not enough to demonstrate any superiority of one analog over the other, but overall, they showed an increased risk of stroke-related outcomes in patients with LBW (HR 1.50 [CI 1.17, 1.92]). Notably, LBW patients in the individual studies of our meta-analysis were mainly on the reduced doses rather than the standard dose compared to non-low body patients. How this influenced the safety and efficacy outcomes is not immediately apparent, and whether it could drive recommendations to commence those patients on standard doses still needs to be determined. In the various studies, reduced doses of direct oral anticoagulants (DOACs) were utilized. These reduced doses included Apixaban 2.5 mg twice daily [23, 33, 35], Rivaroxaban 10 mg [24, 36], Dabigatran 110 mg twice daily [36], and Edoxaban 30 mg [35, 36].

The variation in the prevalence of comorbidities between patients with low body weight (LBW) and those without low body weight may partly explain the differences in observed outcomes. To illustrate, in the study by Boriani et al. [34], LBW patients were noted to be older, frailer, had lower creatinine clearance, and had higher CHA2DS2-VASc scores compared to the normal weight group. Whereas in the study conducted by Hohnloser et al. [33], patients in the low-weight group were more likely to be female, of Asian or Latin American ethnicity, and had a history of stroke, transient ischemic attack, or systemic embolism, as well as abnormal renal function. Additionally, Kadosaka et al. [35] reported that LBW individuals had a higher prevalence of paroxysmal AF and heart failure, with a lower prevalence of antiplatelet usage when compared to individuals with higher body weight. These varying comorbidities and patient characteristics might influence the outcomes in this cohort of patients.

All the DOACs are excreted by the kidney to some extent, which has raised concerns regarding their usage and the need for dose adjustments in individuals with chronic kidney disease (CKD). However, the utilization of DOACs in CKD patients appears to be both safe and effective, especially in those with mild-to-moderate CKD. In a 2019 meta-analysis encompassing 45 trials and involving 34,000 patients, the majority of whom had atrial fibrillation, it was demonstrated that DOACs provided a statistically significant benefit over warfarin in terms of reducing the risk of stroke in atrial fibrillation patients with mild-to-moderate kidney impairment, without an apparent increase in bleeding [49]. A 2020 systematic review, included individuals with atrial fibrillation or venous thromboembolism (VTE) and who had CKD or were undergoing dialysis, showed that DOACs had similar efficacy when compared to warfarin. Additionally, it reported similar bleeding risks when Apixaban was compared to Warfarin [50].

In the ARISTOTLE study, Apixaban was compared to warfarin for stroke reduction in AF patients [51]. During this study, the dosage of Apixaban was adjusted from 5 mg to 2.5 mg for patients with a serum creatinine concentration exceeding 133 mmol/L and who were either over 80 years of age or weighed less than 60 kg. This study showed that Apixaban was more effective than warfarin in reduction of strokes and all-cause mortality and had a lower risk of major bleeding irrespective of the age of the patients.

DOACs are lipophilic drugs, and the anticoagulant effectiveness of these drugs may be affected by body weight due to variations in drug distribution throughout the body. In a pharmacokinetic study [52], Apixaban demonstrated a 27% increase in the mean maximal plasma concentration (Cmax) and a 20% increase in the area under the curve, in underweight patients compared with normal body weight patients. The time to reach maximum Apixaban plasma concentration (tmax) was similar in both weight groups. Additionally, the renal clearance of Apixaban does not exhibit any correlation with an individual’s body weight. Furthermore, the apparent volume of distribution was 14% lower for the low body weight group, as compared with the normal weight group. Variability in hepatic clearance among individuals with extreme body weights, in contrast to those within the normal weight range, might be partly attributed to differences in weight. This is because liver weight, enzyme levels, and metabolic rate are known to correlate with an individual’s body size. Consequently, these factors may help account for the decreased hepatic clearance observed in patients with lower body weights.

A recently published pragmatic, multicenter, open-label, randomized controlled superiority trial conducted on older AF frail patients aimed to investigate the outcomes of switching from INR-guided VKA (vitamin K antagonist) treatment to DOACs compared to continuing VKA treatment. This study showed that switching INR-guided VKA treatment to a NOAC was associated with higher incidence of bleeding complications compared to continuing VKA treatment, without reduction in thromboembolic complications [53]. Our meta-analysis results diverge somewhat from the outcomes observed in the previously mentioned study. These discrepancies may be attributed to variances in the populations being studied, including age, associated comorbidities, active cancer, concurrent antiplatelets, and BMI. For instance, the FRAIL study had an average BMI of around 27, while our analysis compared non-low body weight patients to those with low body weight, defined by a BMI less than 18 or a weight below 60 kg. These distinctions in population characteristics being studied might in part contribute to the disparities in the outcomes.

Limited published meta-analyses have explored the efficacy and safety of DOACs in LBW patients with most studies including patients prescribed either DOAC or warfarin, in contrast to our study which comprises patients exclusively prescribed DOAC for stroke prevention. One such meta-analysis by Grymonprez et al. reported significantly lower risks of stroke/systemic embolism and major bleeding in DOAC-treated patients compared to those on warfarin, with no significant difference in all-cause mortality [54].

In a meta-analysis by Boonyawat et al., a mixed cohort of patients with AF and acute venous thromboembolism (VTE) patients was initiated on either DOAC or warfarin cohort for stroke prevention or VTE treatment. It was found that thromboembolic events occurred more frequently in LBW patients (4.28%) compared to non-LBW patients (2.74%), indicating a potential association between LBW and a higher risk of thromboembolic events in anticoagulated patients, with no significant difference in bleeding outcomes between the LBW group and non-LBW group (5.96% vs. 6.08). Furthermore, subgroup analysis conducted among DOAC-treated patients showed no significant difference in bleeding outcomes between LBW and non-LBW patients, which is consistent with our study’s findings [55].

The significantly higher proportion of composite outcomes among patients with LBW on DOACs emphasizes the stability of the point estimate of individual outcomes tested in our review. This additionally suggests these findings are rather “organic” and unlikely to be attributable to chance alone. The combination of these findings from our meta-analysis resolves some of the lingering uncertainties regarding the exact relationship between patients with LBW following DOAC exposure and hard clinical endpoints. It is also alerting Healthcare professionals about the potential risk of off-label use of DOACs in LBW patients due to enhanced risk of stroke and composite outcomes. Additionally, it suggests further confirmatory attempts by way of prespecified randomized control clinical trials to test these hypotheses.

### Strength and Limitations

Our review represents the first pooled evaluation of currently published papers evaluating uncertainty regarding the exact relationship between exposure to various DOAC analogs in patients with LBW, and our finding from this meta-analysis for the first time provides some level of guidance and potential hypothesis for confirmatory validation of this finding. Additionally, our confirmation of the earlier suggested sequelae of inappropriate underdosing and overdosing for the first time calls for the reclassification of DOACs analogs among patients with LBW as narrow therapeutic index drugs. Our meta-analysis was further constrained by the diversity in the type and dosage of DOACs used in the individual studies.

Previous meta-analytical syntheses, especially those examining the effect of high body weights, were limited by high I2 (denoting high heterogeneity among the constituent studies). We found no such limitations as the I2 point estimate among the three clinical endpoints we evaluated were within the “ballpark” of what is expected for reasonable heterogeneity.

As with all secondary data scheme evaluations, our metanalysis is limited by the usual limitations associated with such data pools, including missing data and lack of uniformity in the adjudication process of various hard clinical endpoints.

## Conclusion

In a pooled examination of primary studies in patients treated with various DOAC analogs, we found increased risk of strokes, overall mortality, and composite outcomes among patient cohorts with low body weight; however, there was no difference in major bleeding episodes. Hence, we stress the significance of vigilant monitoring for patients with low body weight (LBW) and elderly frail individuals who are prescribed DOACs for stroke prevention, due to an elevated risk of thrombotic complications. Moreover, we prioritize patient education to encourage them to promptly seek medical guidance if they experience any new symptoms while on DOAC therapy. Furthermore, this calls for review and incorporation of this reassuring outcome in the DOAC treatment algorithms of national and international treatment guidelines, as the current dose adjustment schemes in light of our findings are inadequate.

## Declarations

### Research Involving Human and Animal Participants

Consent not applicable to this review. However, all authors consented and approved the final manuscript for publication.
